# Supplementary figures and images for: Filter-based models of suppression in retinal ganglion cells: Comparison and generalization across species and stimuli
Source: PLoS Comput Biol. 2025 May 2;21(5):e1013031. doi: 10.1371/journal.pcbi.1013031 (PMC12091892; doi:10.1371/journal.pcbi.1013031)

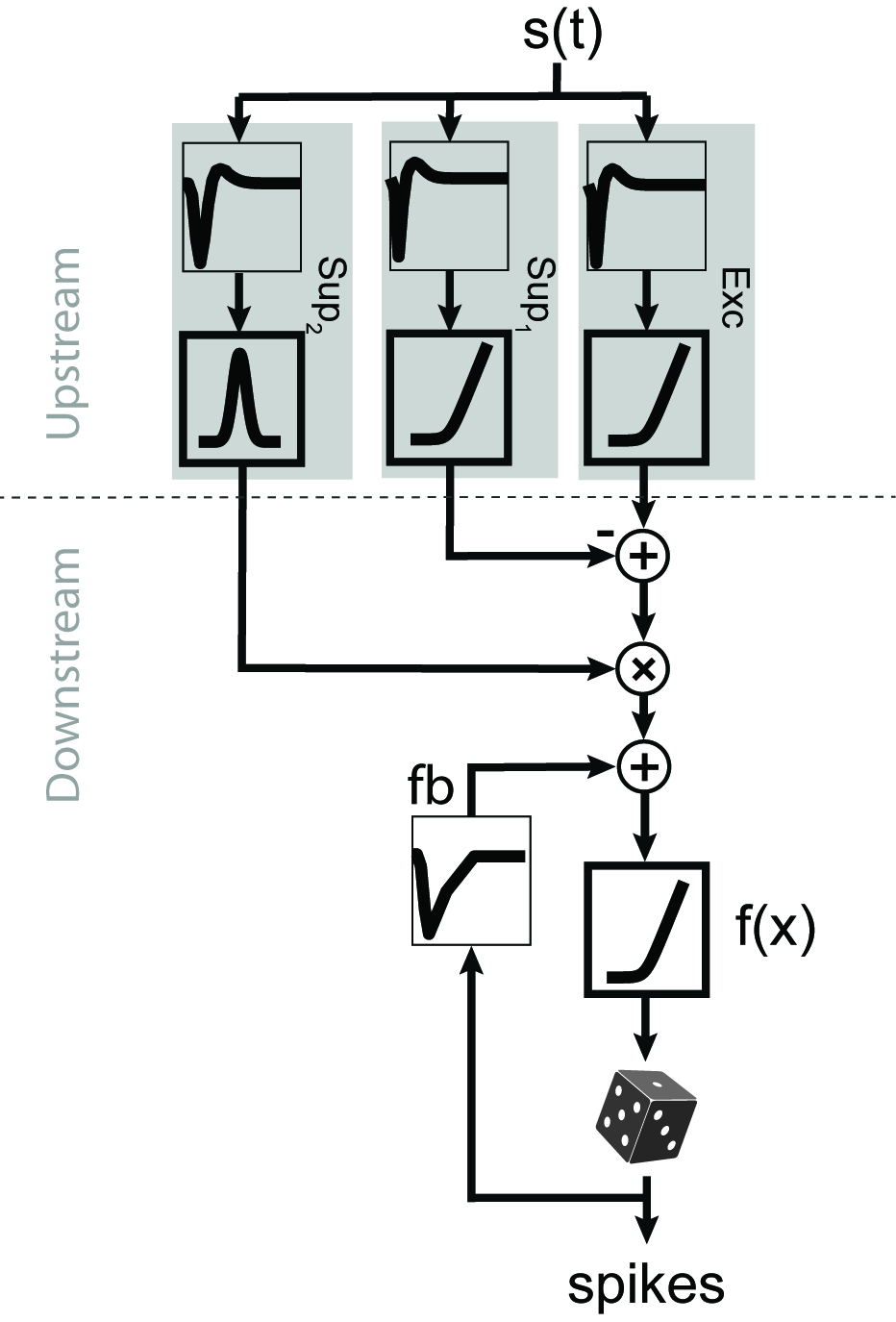

Supplement: S1 Fig — The filter components are shown with thin frames and the nonlinearities with thick frames. Exc upstream branch represents the excitation, Sup1 represents the subtractive suppression, and Sup2 represents the divisive suppression. The feedback (fb) was added to the downstream part of the model. (TIF) [file pcbi.1013031.s001.tif]

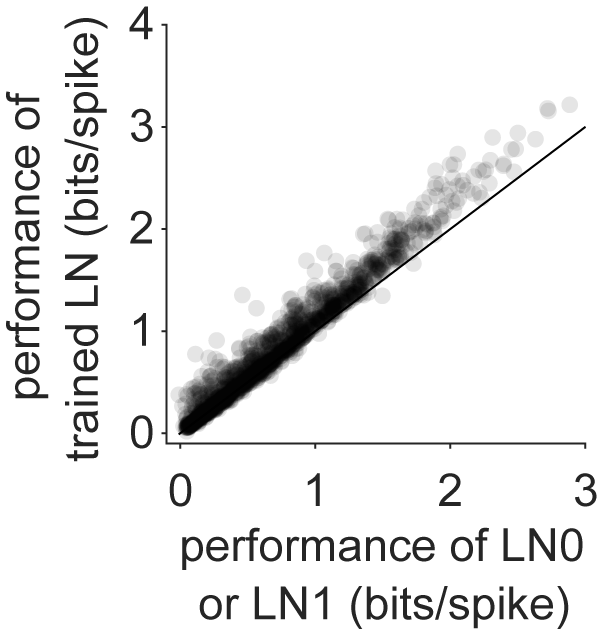

Supplement: S2 Fig — For both LN0 and LN1, the nonlinear function was computed as the histogram of filter outputs versus spike counts. For the comparison here, either LN0 or LN1 was chosen, depending on which model had better performance. (TIF) [file pcbi.1013031.s002.tif]

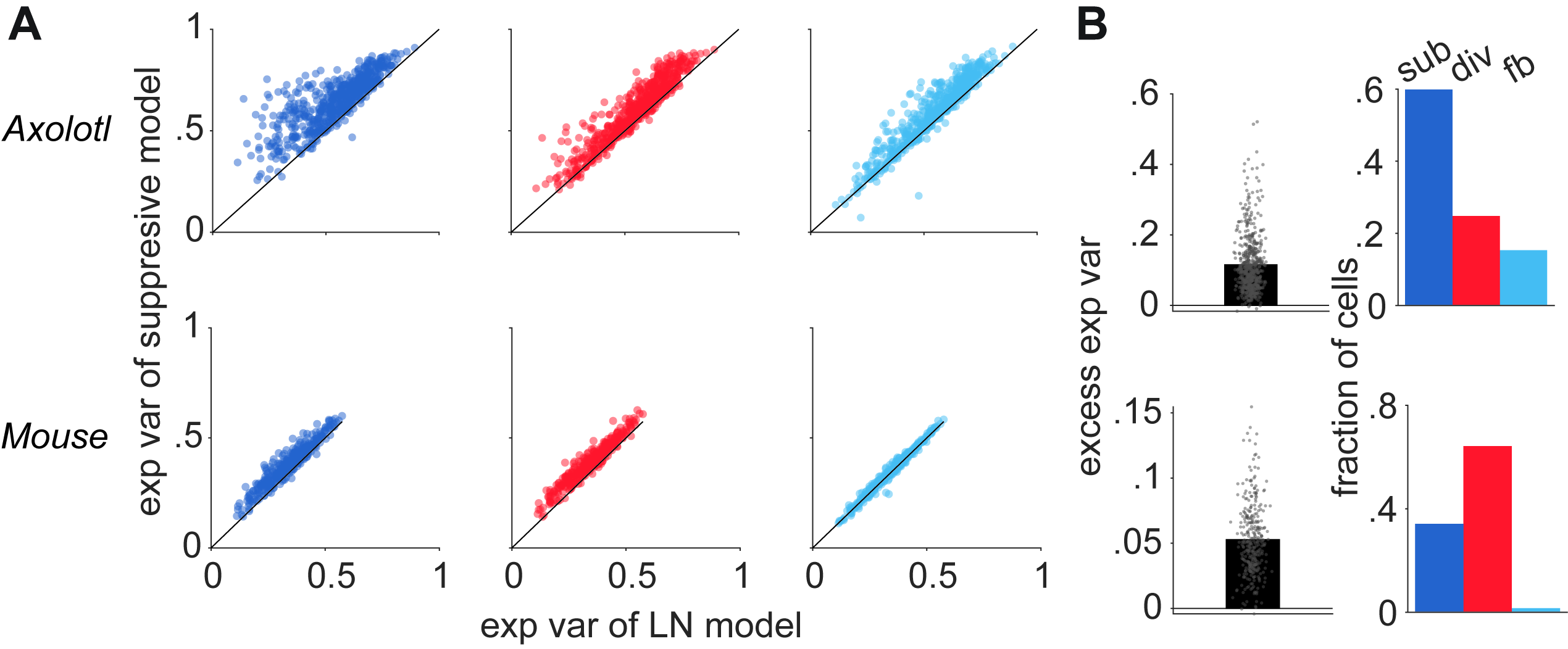

Supplement: S3 Fig — A) Comparison of the Poisson explained variance of each model to the explained variance of the LN model. Each data point represents one cell. B) Left: The excess explained variance (computed as explained variance of suppressive model minus explained variance of the LN model) of the best performing suppressive model, determined for each cell. Right: The percentage of cells for which the corresponding suppressive model outperformed all other models in terms of explained variance. (TIF) [file pcbi.1013031.s003.tif]

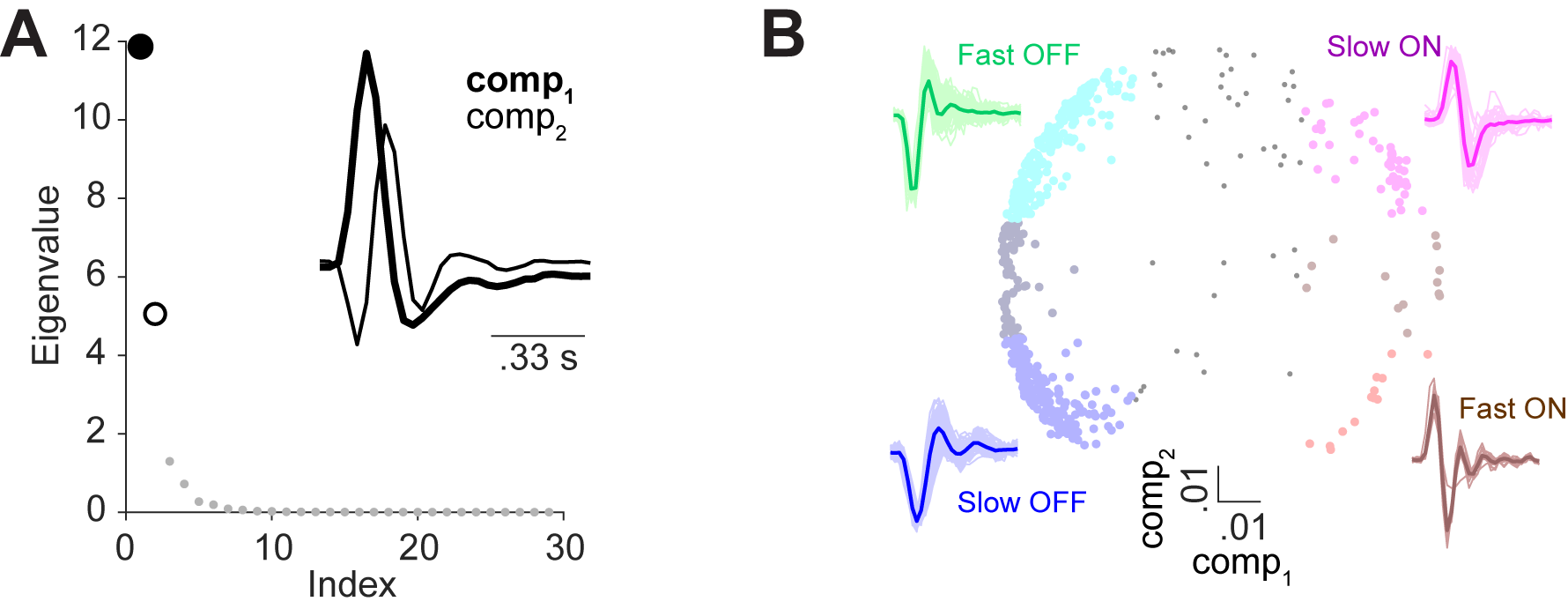

Supplement: S4 Fig — A) The eigenvalues associated with the 30 principal components of the set of filters of the LN model. Inset: The first and the second principal components, which were used for classification. B) Center: All analyzed axolotl cells in the space of s1, the score for the first principal component (comp1), and s2, the score for the second principal component (comp2). To determine the classes, the scores for each cell were compared to thresholds of ±0.02: for slow ON cells (n = 48) s1 > 0.02 and s2 > 0.02, for fast ON cells (n = 13) s1 > 0.02 and s2 < -0.02, for slow OFF cells (n = 195) s1 < -0.02 and s2 < -0.02 and for the fast OFF cells (n = 208) s1 < -0.02 and s2 > 0.02. 143 cells remained unclassified, as they did not pass any of the threshold combinations. Insets surrounding the scatter plot: LN-model filters (thin lines) for each of the identified groups, together with their average (thick line). (TIF) [file pcbi.1013031.s004.tif]

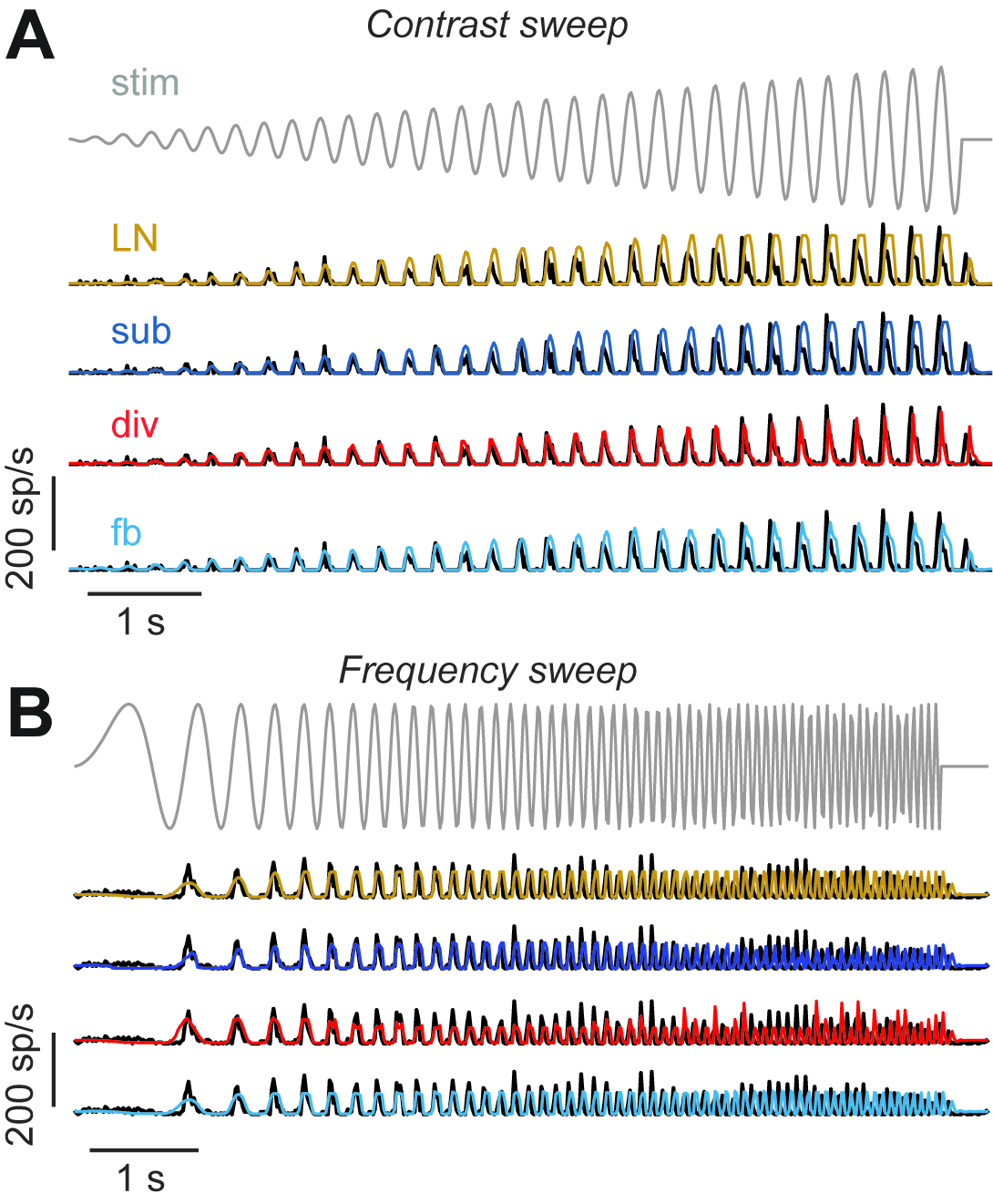

Supplement: S5 Fig — The stimulus traces (gray lines on top) are displayed with temporal sampling corresponding to the monitor update. A) The cell from Fig 5A and 5B for the contrast sweep. B) The cell from Fig 5C and 5D for the frequency sweep. (TIF) [file pcbi.1013031.s005.tif]
