# Supplementary material for: Filter-based models of suppression in retinal ganglion cells: Comparison and generalization across species and stimuli
Source: PLoS Comput Biol. 2025 May 2;21(5):e1013031. doi: 10.1371/journal.pcbi.1013031 (PMC12091892; doi:10.1371/journal.pcbi.1013031)
Supplement: Attachment — Submitted filename: point_by_point_response.pdf [file pcbi.1013031.s007.pdf]

We thank the Reviewers for their constructive comments. We have addressed all aspects raised by the Reviewers as detailed in the point-by-point responses below. In particular, we have extended the mechanistic and functional interpretation and discussion of our findings, with emphasis on the differences between the subtractive and the divisive models. We have also considerably strengthened the links to and the discussion of previous works on suppression models. To allow tracking the revisions and the new material, we have provided both a version of the manuscript where changed and added text is highlighted as well as a clean copy of the revised manuscript.

***Reviewer #1:***

*In this study the authors compare the performance of several models of suppression at explaining quantitatively how ganglion cells respond to random fluctuations of contrast, for a stimulus that is spatially uniform. They find that suppressive and divisive models are better than a spike history filter model. This is a solid study with interesting results.*

*My concerns:*

*1) My understanding is that the feedback model is just implementing the kind of spike history filter usually found in GLM models (Pillow et al). There are other models that perform at this, and have been used before. For example, the gain control model used in Berry et al, 1999, but also in Chen et al, 2013,2014 ; Leonardo and Meister, etc. This gain control model has a divisive component. It seems necessary to compare the performances to this type of model too.*

Thanks for bringing this up. Indeed, the feedback model that we consider is in the style typically applied as GLMs, and we now point this out more clearly when introducing the model. Furthermore, we now take up the feedback model as introduced by Berry et al 1999 and show in the Discussion that it can be viewed (to good approximation) as a special case of the divisive model of our paper. We therefore decided not to include a direct comparison with this model, but rather explain the structural and functional relation of the Berry et al model to the models investigated in our work and point out that the true strength of the Berry et al model arguably lies in its application to spatiotemporal stimuli which are not part of the present work. These aspects are now contained in a new section on “Relation to previous work” in the revised Discussion.

*2) The main result is that the divisive and the subtractive models are not doing exactly the same thing. Their performance depend on stimulus frequency, a result I find very interesting. It would be nice to have more intuition about why this is the case.*

Indeed, the differences between the divisive and subtractive model and their different regimes of best performance are key results of our work, and we have strived in the revision to provide better insight and more intuition about this result. In particular, we now use the examples of Figure 3, in particular the slow and fast axolotl cells, which are best captured by the subtractive and divisive model, respectively, to illustrate how the subtractive model manages to capture small response peaks by modulating suppression (while divisive suppression can do little during these periods when excitatory input is low or zero) and how the divisive model manages to better shape (large) response peaks by curbing the trailing edges of the peaks. In the Discussion, we have added a new section on “Functional interpretation” where we pick up this observation again and also relate it to how the two models differ in their performance regimes under contrast sweeps and frequency sweeps.

*3) A caveat of this study, acknowledged by the authors, is that everything they do is restricted to spatially uniform stimuli. The lack of substantial difference between the divisive and subtractive model could come from this. I don’t expect the authors to address the issue of spatially detailed stimuli, but a bit more discussion on this would be helpful, especially given the other works in the*

*field.*

We agree that an extended discussion of the limitation to spatially homogeneous stimuli is important for relating our findings to the case of spatiotemporal stimulation. As suggested, we now elaborate on this aspect in the partly new section on “Model complexity and limitation to spatially homogeneous stimuli” in the Discussion where, in particular, we point out the relevant aspect of capturing suppression both from the center and the surround region of the receptive field and relate our work to examples in the literature.

*4) Related to this, the discussion contains very little discussion about other works in the field (eg ref 1 of the paper). This is an issue to better identify the novelty of this work.*

We have now considerably extended the discussion of how our findings relate to other works in the field, both with respect to spatiotemporal stimuli (see point 3 above), as well as more generally throughout the text and in particular in a new section on “Relation to previous work” in the Discussion (see also point 1 above).

*5) Some parts of the paper are a bit unclear. In particular, the paragraph that includes the sentence: In the divisive model, on the other hand, the shape of the nonlinearity in the suppressive branch led to a unilateral, rectified suppression with essentially no sensitivity to filter activations near to or smaller than zero is hard to understand for me and deserves a proper explanation. The first panel of the figure 3 might benefit from being expanded. The non-linearity is learned but this is not obvious from the text. Also, the NL learning seems to hit the bounds imposed. What happens if they are pushed back ?*

Thanks for pointing this out. Indeed, our explanation here has been a bit unclear, and the revision allowed us to reformulate our explanation regarding the different behaviors of the models for clarification and for connecting it better to the subtractive and divisive nature of the suppression. This concerns particularly the part of the Results where Fig. 3A,B are described and discussed, but we have strived for similar clarifications also elsewhere in the manuscript. The revised text should also make it clear now that it is not primarily the constraints on the nonlinearities that cause the different behaviors of the subtractive and divisive model, but rather the subtractive and divisive nature of the models. The particular constraints on the nonlinearities have been chosen to be consistent with previous usage in the literature and to maintain interpretability of the model branches as excitatory and suppressive. For example, the constraint on the nonlinearity of the divisive model branch is not easily relaxed without allowing it to act as an excitatory component. This is now explained better in the Methods section. We have also expanded panels A-D of Fig. 3 as suggested. In panel A, in particular, important details should now be easier to discern.

***Reviewer #2:***

*This is a thorough and well-executed exploration of different classes of combined excitation and suppression in neurons. The work focuses on retinal ganglion cells and taps three species (axolotl, mouse, marmoset) to add a comparative angle to the study. Models are well-characterized and compared for two main classes of input stimuli - full-field flicker, chirps, and contrast sweeps. Results show that divisive suppression is the most versatile motif, with good reproduction of responses across the widest range of stimuli. Subtractive suppression was only the clear winner in slow axolotl cells. A large number of recordings were made, the methods are sound and well-explained, and the text flows well and is well-referenced.*

*Major comment:*
*The only way this paper could be found lacking is in a clearer or more thoroughly explored functional*

*consequence to the findings. The Discussion certainly hints at some ideas surrounding the differences in these types of suppression, but more could be made about:*
*1) the differences between axolotl and mouse/primate: is this a result of "more processing" happening in the amphibian retina compared to mammalian species? Does this connect to behavioral repertoire or environmental statistics in some potentially interesting way?*
*2) how divisive suppression might be better suited to dynamic environments. Some of the Discussion touches on adaptive mechanisms, but a bit more could be said here.*

Thanks for these suggestions. We now consider the functional implications of our findings in more detail in a new section on "Functional interpretation" in the Discussion. In particular, we highlight how divisive suppression might be particularly suited to shape strong spiking events for fast stimuli by curbing the trailing edge, owing to the effectiveness of divisive suppression at high levels of excitation. This is also supported by a more detailed discussion of the differences between the two models in Fig. 3. Regarding the difference between axolotl and the other two species in our work, we do not see an easy connection to environmental statistics or behavior, but we now hypothesize that this is connected to the frequently observed sparsity of spiking events in the amphibian retina and the relatively slow kinetics of their retinal responses. These aspects are also contained in the new section on "Functional interpretation".

*This points could better expand the reach of the work beyond the retinal community, though the work stands as a useful and solid set of insights for cortical neurophysiologists. A more explicit connection to non-retinal processing in the Discussion could be useful.*

Thanks for bringing this up. As suggested, we now better connect our work to studies beyond the retina, in particular regarding the investigation of divisive normalization and the distinction between subtractive and divisive suppression in primary visual cortex. This can be found in the revised manuscript in a new section "Beyond the retina" in the Discussion.

*Minor comment:*
*On page 10, is "motives" meant to be "motifs"?*

Indeed, thanks for catching this. Now corrected.
